# Estimating numerical error in neural network simulations on Graphics Processing Units

**DOI:** 10.1186/1471-2202-16-S1-P182

**Published:** 2015-12-18

**Authors:** James P Turner, Thomas Nowotny

**Affiliations:** 1 Centre for Computational Neuroscience and Robotics, University of Sussex, Brighton, UK

## 

**Figure 1:**
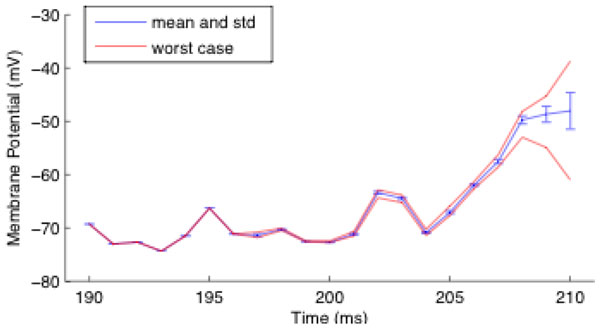
**In repeated runs, results of numerical simulations on GPUs can vary**. Mean, standard deviation and range of observed membrane potential of a neuron in a network of 10,000 Izhikevich neurons, 8,000 excitatory and 2,000 inhibitory, with 1,000 random connections each; after only 190 ms simulation the results start to diverge visibly, and after only 210 ms, they differ largely.

Modern graphics processing units (GPUs) are becoming a popular hardware substrate for spiking neural network simulations [1-3], due to their massive parallelism and impressive cost-to-speed ratio. However, verifying and interpreting the results of a GPU simulation can be difficult because the results are never exactly reproducible, unlike those from an equivalent serial simulation on a CPU; not only is the simulation subject to the usual rounding errors of floating-point arithmetic, but there are also elements of stochasticity due to the non-determinism of the thread scheduling mechanism on the hardware, alongside the non-associativity of floating-point addition and multiplication. Consider, for example, a typical postsynaptic integration step for the summation of incoming synapse currents, executed on a GPU. If there are multiple threads, which are each simulating an incoming synapse, there is no guarantee for the order in which each synapse thread's current will be accumulated into the total current. Therefore, rounding errors will be different depending on this order and the result could be different every time the simulation runs. Such effects would initially be small but can be amplified in unstable or chaotic systems to a degree that the final results appear completely random across different runs (see Figure 1).

When comparing runs between GPU and CPU implementations there are additional sources of divergence. There are subtle differences in the way each architecture implements floating-point arithmetic. For instance, the NVIDIA C2070 GPU tested in this study implements the fused multiply-add (FMA) operation, introduced in the latest IEEE-754-2008 floating-point standard, whereas most Intel CPUs perform the multiplication and addition operations separately, with lower accuracy. Only Intel's most recent Haswell CPU architecture implements the more accurate FMA operation, whilst many current lab workstations contain chips that do not.

The aim of the work presented here is to analytically determine the theoretical worst-case and average-case numerical absolute error incurred when simulating neural network models on an NVIDIA CUDA GPU. These error measurements are also compared with the absolute error resulting from the equivalent serial algorithm running on a single CPU core, using standard float32 (float) and float64 (double) precision floating-point arithmetic, to determine a reasonable error margin for verifying the results of parallel GPU simulations against those of equivalent serial CPU simulations. Furthermore, both CPU and GPU implementations are compared against an equivalent simulation using an accurate arbitrary-precision floating-point arithmetic library, to determine how far the CPU and GPU simulation trajectories deviate from the analytically 'correct' trajectory. For illustration, the divergence of a single neuron in a 10,000 neuron Izhikevich network is plotted in the figure. Finally, we also analyse the role of errors originating from approximate integration methods and compare them to the underlying numerical errors discussed thus far.
